# Reduced somatostatin signalling leads to hypersecretion of glucagon in mice fed a high-fat diet

**DOI:** 10.1016/j.molmet.2020.101021

**Published:** 2020-05-21

**Authors:** Joely A. Kellard, Nils J.G. Rorsman, Thomas G. Hill, Sarah L. Armour, Martijn van de Bunt, Patrik Rorsman, Jakob G. Knudsen, Linford J.B. Briant

**Affiliations:** 1 Oxford Centre for Diabetes, Endocrinology and Metabolism, Radcliffe Department of Medicine, Churchill Hospital, Oxford OX3 7LE, UK; 2 Section for Cell Biology and Physiology, Department of Biology, University of Copenhagen, Denmark; 3 Department of Bioinformatics and Data Mining, Novo Nordisk A/S, Maaloev, Denmark; 4 Department of Neuroscience and Physiology, University of Göteborg, Box 430, SE40530 Göteborg, Sweden; 5 Oxford National Institute for Health Research, Biomedical Research Centre, Churchill Hospital, Oxford OX3 7LE, UK; 6 Department of Computer Science, University of Oxford, Oxford OX1 3QD, UK

**Keywords:** Diabetes, High fat diet, Islet of Langerhans, Alpha cell, Insulin tolerance, Hyperglucagonemia, Paracrine, Calcium, Somatostatin, Delta cell

## Abstract

**Objectives:**

Elevated plasma glucagon is an early symptom of diabetes, occurring in subjects with impaired glucose regulation. Here, we explored alpha-cell function in female mice fed a high-fat diet (HFD).

**Methods:**

Female mice expressing the Ca^2+^ indicator GCaMP3 specifically in alpha-cells were fed a high-fat or control (CTL) diet. We then conducted *in vivo* phenotyping of these mice, as well as experiments on isolated (*ex vivo*) islets and in the *in situ* perfused pancreas.

**Results:**

In HFD-fed mice, fed plasma glucagon levels were increased and glucagon secretion from isolated islets and in the perfused mouse pancreas was also elevated. In mice fed a CTL diet, increasing glucose reduced intracellular Ca^2+^ ([Ca^2+^]_i_) oscillation frequency and amplitude. This effect was also observed in HFD mice; however, both the frequency and amplitude of the [Ca^2+^]_i_ oscillations were higher than those in CTL alpha-cells. Given that alpha-cells are under strong paracrine control from neighbouring somatostatin-secreting delta-cells, we hypothesised that this elevation of alpha-cell output was due to a lack of somatostatin (SST) secretion. Indeed, SST secretion in isolated islets from HFD-fed mice was reduced but exogenous SST also failed to suppress glucagon secretion and [Ca^2+^]_i_ activity from HFD alpha-cells, in contrast to observations in CTL mice.

**Conclusions:**

These findings suggest that reduced delta-cell function, combined with intrinsic changes in alpha-cells including sensitivity to somatostatin, accounts for the hyperglucagonaemia in mice fed a HFD.

## Highlights

• HFD feeding causes hyperglucagonaemia *in vivo*.
• Glucagon is inadequately suppressed by glucose in HFD-fed mice.
• Alpha-cell [Ca^2+^]_i_ oscillations and glucagon output are elevated *ex vivo* in response to HFD feeding.
• SST secretion from HFD islets is reduced.
• Alpha-cells from HFD-fed mice become ‘resistant’ to SST.

## 1 Introduction

Type 2 diabetes (T2D) is characterised by elevated circulating glucose. Lack of insulin plays an important role in the development of hyperglycaemia and glucose intolerance in T2D. However, it is also recognized that abnormal glucagon secretion contributes to the development of glucose intolerance and that T2D is best characterised as a bihormonal disorder [1,2].

Glucagon is secreted from alpha-cells of the pancreatic islets when plasma glucose falls below ∼4 mM. Glucagon secretion is regulated within the islet by both intrinsic and paracrine mechanisms [3]. Glucose can directly inhibit glucagon secretion, but there is still no consensus about the nature of this intrinsic mechanism(s) [[3], [4], [5], [6], [7], [8], [9], [10]]. In particular, glucose has been proposed to increase the intracellular ATP and that this, via closure of plasmalemmal ATP-regulated K^+^ (K_ATP_) channels, results in membrane depolarization and reduction in action potential height (due to voltage-dependent inactivation of the Na^+^ channels involved in action potential firing). This culminates in reduced activation of voltage-gated Ca^2+^ channels and, consequently, exocytosis of glucagon-containing granules [11]. However, glucose has also been demonstrated to intrinsically inhibit glucagon secretion by a mechanism involving store-operated channels [12] or altered cAMP signalling (see [9] and reviews [13,14]). Glucagon release is also influenced by local paracrine signals. These include somatostatin [15,16] and insulin [17,18] from islet delta- and beta-cells, respectively.

In some patients with T2D, the normal relationship between plasma glucose and glucagon is reversed and hyperglycaemia stimulates rather than inhibits glucagon secretion [11,19]. The dysregulation of glucagon secretion in T2D is detectable even prior to the onset of diabetes; hyperglucagonaemia is observed in obese patients [20,21] and patients with impaired fasting glycaemia [22]. Although it is clear that glucagon is central in the aetiology of T2D, we still do not understand how glucagon secretion is affected by the changes in whole body metabolism that precede the onset of the disease. In particular, the impact of high-fat diet (HFD) feeding—widely regarded as a model of prediabetes [23]—on glucagon secretion is not well characterised. Exposure of whole islets to high levels of palmitate for up to 72 h changes insulin, glucagon, and somatostatin secretion [24,25] as well as the whole-islet gene expression [26] and metabolism [27,28]. Furthermore, isolated islets from HFD mice exhibit elevated glucagon secretion when exposed to high glucose concentrations [29]. However, the mechanism by which this elevation occurs remains unresolved and obscured by the conflicting *in vivo* observations that circulating glucagon is increased [29], decreased [30], or unchanged [31] in HFD mice. Here, we investigate the effects of HFD feeding on alpha-cell function and the paracrine regulation of glucagon secretion.

## 2 Methods

### 2.1 Ethics

Experiments were conducted in strict accordance with the UK Animals Scientific Procedures Act (1986) and the University of Oxford ethical guidelines. All work was approved by the Local Ethical Committee.

### 2.2 Animals

Mice expressing GCaMP3 specifically in alpha-cells were generated by crossing *Gt(ROSA)26Sor*^*tm38(CAG-GCaMP3)Hze*^ mice (Jackson Laboratory No. 014538) with mice carrying an insert containing glucagon promoter-driven iCRE (*Tg(Gcg-icre)*^*12Fmgb*^ mice; see [32]). Heterozygous breeding was set up to produce in mice heterozygous for the *Tg(Gcg-icre)*^*12Fmgb*^ and the *Gt(ROSA)26Sor*^*tm38(CAG-GCaMP3)Hze*^ allele. iCRE was always and only passed down through the father. All mice used in this study were 16–18 weeks old and fully backcrossed to a C57BL/6J background. Given the large differences in body weight, blood glucose, and the response to HFD feeding between sexes, we chose to restrict our study to female mice. Unless otherwise indicated, animals had *ad libitum* access to food and water. All animals were housed in an SPF facility on a 12:12 h light:dark cycle at 22 °C. In all cases where animals fasted, food was removed at 08.30 a.m. (30 min into the light phase). Immediately after weaning, mice were fed either a high-fat (HFD) (% kcal: protein 18.3, carbohydrate 21.4, fat 60.3; TD.06414, Envigo) or a control diet (CTL) (% kcal: protein 20.5, carbohydrate 69.1, fat 10.5; TD.08806 Envigo) for 12 weeks. Mice were cohoused by diet and litters were mixed to avoid litter-specific effects of diet.

### 2.3 Glucose tolerance test

Following 6 h of fasting, animals received an intraperitoneal (i.p.) injection of d-glucose (2 g/kg; IPGTT). Blood glucose concentrations were measured at 0, 15, 30, 60, and 120 min after the injection. A sample was also taken 15 min prior to the injection (“Rest”). Blood samples (25 μL) were obtained by tail vein puncture at 0 and 30 min in EDTA-coated capillary tubes. Whole blood was immediately mixed with 5 μL of aprotinin (1:5, 4 TIU/mL, Sigma–Aldrich, UK) and kept on ice until it was centrifuged at 2600 g at 4 °C. Plasma was then removed and stored at −80 °C.

### 2.4 Fed plasma measurements

Tail vein blood samples were also taken from *ad libitum* fed mice with free access to water, housed in their home cage. Blood samples were taken at 09:00, 13:00, and 17:00 and processed as described previously.

### 2.5 Insulin tolerance test

Following 4 h of fasting, animals received an i.p. injection of insulin dosed on total body weight (0.75 U/kg total body weight; Actrapid, Novo Nordisk). This insulin tolerance test (ITT) involved measuring blood glucose concentrations at 0, 15, 30, 60, and 120 min after the injection. At fixed time points following the injection, 25 μL of blood was obtained and processed as above.

In an additional experiment, mice were given an insulin bolus where the insulin was dosed on lean mass. Initial experiments using EchoMRI™ (EchoMRI LLC, USA) demonstrated that CTL mice were 69.5 ± 2.1% lean mass, whereas HFD-fed mice were 59.5 ± 3.2% lean mass (*P* = 0.023, n = 6 CTL and 6 HFD mice, unpaired *t*-test; Table 1). Therefore, for the lean mass-based insulin injections, CTL mice received 0.75 U/kg total body weight, whereas HFD-fed mice received 0.64 U/kg, thereby giving the mice the same dose of insulin per gram lean mass (1.08 U/kg lean mass).

**Table 1:** Body composition analysis of mice on HFD and CTL diet for 12 weeks by EchoMRI™.

Parameter	CTL (n = 6)	HFD (n = 6)	*P*
Weight (g)	20.77 ± 0.35	26.25 ± 1.69	0.0035
Fat %	20.33 ± 2.46	32.88 ± 4.19	0.0273
Lean mass %	69.50 ± 2.09	59.50 ± 3.24	0.0268
Total water %	57.16 ± 4.42	48.63 ± 5.61	0.0151
Fat (g)	4.21 ± 0.49	8.90 ± 1.59	0.0183

### 2.6 Islet isolation

Mice were culled by cervical dislocation. Pancreatic islets were isolated by liberase digestion followed by manual picking. Isolated islets were, pending the experiments, maintained in short-term (<24 h) tissue culture in RPMI 1640 (11879-020, Gibco, Thermo Fisher Scientific) containing 1% penicillin/streptomycin (1214-122, Gibco, Thermo Fisher Scientific), 10%FBS (F7524-500G, Sigma–Aldrich), and 11 mM glucose prior to the measurements.

### 2.7 Static secretion experiments

Islets isolated from HFD and control mice were incubated in 11 mM glucose media. All secretion experiments were conducted on the day of islet isolation, following 1–2 h culture. Size-matched batches of 20 islets were then preincubated in 0.2 mL KRB (in mM; 140 NaCl, 5 KCl, 1.2 MgCl_2_, 2.6 CaCl_2_, 1 NaH_2_PO_4_, 5 NaHCO_3_, and 10 HEPES (pH 7.4)) with 2 mg/mL BSA (S6003, Sigma–Aldrich) and 3 mM glucose for 1 h at 37 °C. Following this, islets were subjected to 1 mM or 6 mM glucose KRB with 0.2% BSA for 1 h. The supernatant was removed, quickly frozen, and stored at −80 °C. For the measurement of total glucagon and insulin contents, the islets were lysed in HCl:ethanol (1:15) at the end of the experiment, sonicated and stored at −80 °C.

### 2.8 The *in situ* perfused mouse pancreas

Briefly, the aorta was ligated above the coeliac artery and below the superior mesenteric artery and then cannulated. The pancreas was perfused with KRB containing varying concentrations of glucose and somatostatin-14 (Tocris, Cat. No 1157) as indicated in the figures, at a speed of 1.34 μL/min/mg pancreas weight using an Ismatec REGLO Digital MS2/12 peristaltic pump. Pancreatic weight was estimated from the whole body weight as previously described [33,34]. The perfusate was maintained at 37 °C using a Warner Instruments temperature control unit TC-32 4B in conjunction with an in-line heater (Warner Instruments P/N 64-0102) and a Harvard Apparatus heated rodent operating table. The effluent was collected in intervals of 1 min into 96-well plates which were kept on ice and contained aprotinin. Samples were subsequently stored at −80 °C pending analysis of glucagon content.

### 2.9 Hormone measurements

Plasma insulin and glucagon were determined using insulin and glucagon mouse sandwich ELISA (10-1113-01 and 10-1281-01 from Mercodia, Sweden). Insulin and glucagon concentrations from *ex vivo* islet experiments were measured using mouse/rat insulin-glucagon sandwich ELISA (K15145C, Mesoscale Discovery, USA), and somatostatin concentration was determined using radioimmunoassay (Life Science AB, Sweden). Glucagon concentrations from the perfusate of the *in situ* perfused mouse pancreas were measured using the U-plex Glucagon ELISA (K1515YK, Mesoscale Discovery). All measurements were conducted according to the manufacturers’ protocols.

### 2.10 GCaMP3 imaging and calculation of [Ca^2+^]_i_ spike frequency and amplitude

Time-lapse imaging of the intracellular GCaMP3 was performed on the inverted Zeiss AxioVert 200 microscope, equipped with the Zeiss LSM 510-META laser confocal scanning system, using a 40×/1.3 NA objective. The chamber was continuously perfused at a rate of 200 μL/min with KRB solution (described above) containing 2 mg/mL BSA (S6003, Sigma–Aldrich), glucose, and other compounds as indicated in the figures. All solutions were corrected for osmotic differences with mannitol. GCaMP3 was excited at 488 nm and fluorescence emission was collected at 530 nm at a frequency of 1.28 Hz. Fiji (http://fiji.sc/Fiji) was used to identify and measure the intensity of the GCaMP3 signal in individual regions of interest (cells) over time. Given that the specificity of CRE is not 100%, we expected a number of GCaMP3^+^ cells to be non-alpha-cells. Therefore, only GCaMP3^+^ cells that (a) were active at 1 mM glucose and (b) exhibited an increase in Ca^2+^ in response to adrenaline (5 μM) in the presence of high glucose (15 mM; see [35]) were considered alpha-cells. This process consistently resulted in the removal of ∼10% of the GCaMP3^+^ cells, in keeping with the specificity of this iCRE line that we report here (as outlined in Figure 5A and the associated text in the Results section). Spikes in GCaMP3 were manually annotated using Spike2 (http://ced.co.uk/) and defined as being spikes if their amplitude was >20% the amplitude of a period of noise. Spike frequency was calculated from this annotated data, and spike amplitude was calculated from the spike-triggered average of the fluorescence signal over a fixed time window (10 time-steps).

### 2.11 Perforated patch-clamp recordings

Islets isolated from chow-fed as well as CTL and HFD mice were used for electrophysiological recordings. These recordings (in intact islets) were performed at 33-34 °C using an EPC-10 patch-clamp amplifier and PatchMaster software (HEKA Electronics, Lambrecht/Pfalz, Germany). Unless otherwise stated, recordings were made in 3 mM glucose. Currents were filtered at 2.9 kHz and digitized at > 10 kHz. A new islet was used for each recording. Membrane potential (V_m_) recordings were conducted using the perforated patch-clamp whole-cell technique as previously described [36]. The pipette solution contained (in mM) 76 K_2_SO_4_, 10 NaCl, 10 KCl, 1 MgCl_2_·6H_2_0, and 5 HEPES (pH 7.35 with KOH). For these experiments, the bath solution contained (mM) 140 NaCl, 3.6 KCl, 10 HEPES, 0.5 MgCl_2_·6H20, 0.5 Na_2_H_2_PO_4,_ 5 NaHCO_3_, and 1.5 CaCl_2_ (pH 7.4 with NaOH). Glucose was included in the extracellular medium at the indicated concentrations. Amphotericin B (final concentration of 25 mg/mL, Sigma–Aldrich) was added to the pipette solution to give electrical access to the cells (series resistance of <100 MΩ). Alpha-cells were confirmed by the presence of GCaMP3 or RFP. In WT islets, alpha-cells were identified by the presence of action potential activity at 3 mM glucose and ion channel properties [37]. In some recordings, GCaMP3 was also simultaneously recorded with a Hamamatsu ORCA 2, operated with MicroManipulator.

The frequency of action potential firing was calculated in MATLAB v. 6.1 (2000; The MathWorks, Natick, MA, USA). In brief, a peak-find algorithm was used to detect action potentials. This was then used to calculate firing frequency and correlate average firing frequency (calculated every 2 s) with the GCaMP3 signal.

### 2.12 Immunofluorescent staining

Whole pancreases were harvested and fixed in 4% PFA for up to 24 h before embedding in wax; 5-μm-thick sections were cut and stained using the antibodies indicated below.

**Table:** 

Ig (primary)	Ig (secondary)
Chicken anti-GFP (Life Technologies, A10262)	Goat anti-chicken 488 nm (Invitrogen, A11029)
Mouse anti-glucagon (Sigma–Aldrich, G2654)	Goat anti-mouse 568 nm (Invitrogen, 11032)
Guinea pig anti-insulin	Goat anti-guinea pig 633 nm
Rabbit anti-somatostatin	Goat anti-rabbit 488 nm
Rabbit anti-SSTR2 (Abcam, ab134152)	Vector Labs DyLight Kit (DK-1488)

Stained sections were imaged using a BioRad Radiance 2000 Scanning Laser Confocal Microscope.

Primary and secondary antibodies used for the staining of mouse pancreases. All dilutions are 1:500.

### 2.13 Statistics

All data are reported as mean values ± SEM unless otherwise stated. Statistical significance was defined as *P* < 0.05. All statistical tests were conducted in Prism 8.0 (GraphPad Software, San Diego, CA, USA). For two groupings, a *t*-test was conducted. A Mann–Whitney test was conducted for data not normally distributed. For more than two groupings, a one-way ANOVA was conducted. If there were two independent variables, a two-way ANOVA was conducted. If the data passed normality criteria (D'Agostino's test of normality and Bartlett's test of equal variances), a parametric test was conducted with the appropriate post hoc test (Tukey or Sidak). If the normality criteria were not met, a Kruskal–Wallis test with Dunn's multiple comparison test was conducted.

## 3 Results

### 3.1 HFD alters glucose homeostasis and plasma glucagon concentration *in vivo*

**Figure 1:**
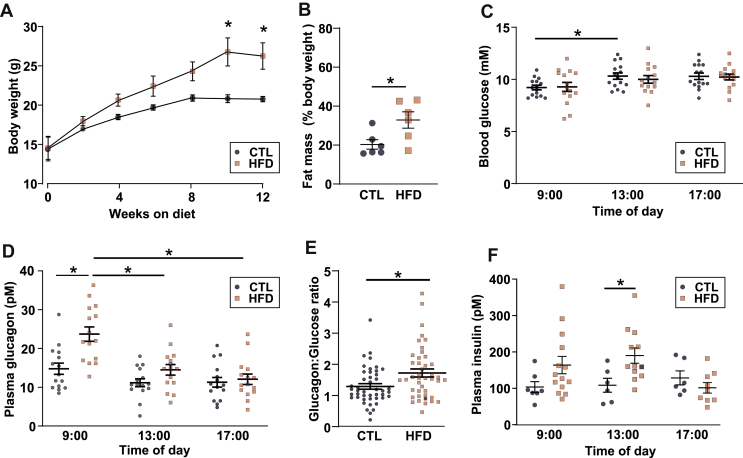
**HFD feeding evokes hyperglucagonaemia *in vivo.*** A. Increase in bodyweight in response to high-fat diet (HFD) or control diet (CTL). Two-way repeated measures ANOVA; ∗*P* < 0.05. Data are presented as mean ± SEM. B. Total body fat as % of total body weight in response to CTL or HFD at 12 weeks on diet. Unpaired *t*-test; ∗*P* < 0.05. Data are presented as mean ± SEM. C. Fed blood glucose during the day for mice on the CTL (N = 15) or HFD (N = 14). Two-way repeated measures ANOVA; ∗*P* < 0.05. Data are presented as mean ± SEM. D. Same as C but plasma glucagon. Two-way repeated measures ANOVA; ∗*P* < 0.05. E. The glucagon:glucose ratio for mice in the CTL and HFD. Ratios are calculated from all 3 time points from C and D. Unpaired *t*-test; ∗*P* < 0.05. Data are presented as mean ± SEM. F. Same as C but plasma insulin, CTL (n = 6–7 mice) and HFD (n = 9–14 mice). Two-way repeated measures ANOVA; ∗*P* < 0.05. Data are presented as mean ± SEM.

Following weaning, female mice were fed either a HFD (60% dietary calories from fat) or a CTL diet (10% calories from fat) for 12 weeks. HFD feeding resulted in an increase in body weight and fat mass (Figure 1A and B and Table 1). To determine whether HFD feeding affected glycaemia, we measured blood glucose and plasma glucagon in *ad libitum* fed animals over several time points during the light phase. Blood glucose was not different between CTL and HFD mice at any time (Figure 1C). Despite this, HFD mice had higher plasma glucagon levels than CTL mice at the beginning of the light phase (09:00 am; Figure 1D). We analysed the glucagon:glucose ratio from all time points and found that it was higher in HFD mice (Figure 1E), supporting the notion that the relationship between glucagon and glucose was altered in HFD mice. Insulin is a known paracrine inhibitor of glucagon [2,17,18], but the levels of circulating insulin were in fact elevated in response to HFD feeding (Figure 1F), making it unlikely that the elevated plasma glucagon in HFD mice was secondary to reduced plasma insulin.

**Figure 2:**
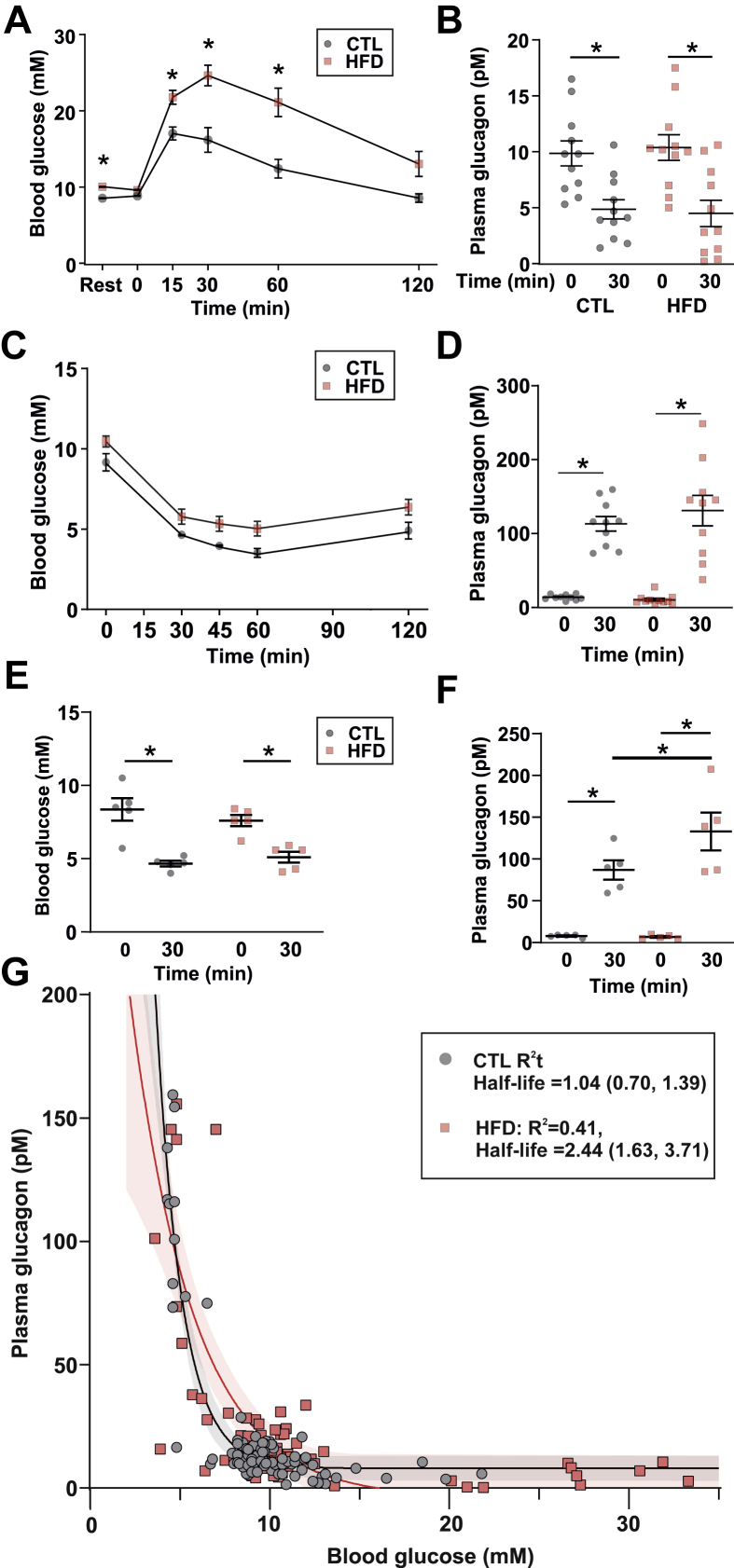
**Counterregulatory glucagon secretion is elevated in response to HFD feeding.** A. Blood glucose of mice in response to an i.p. GTT (2 mg/kg) in mice on a CTL (n = 12) or HFD (n = 13) diet. Two-way repeated measures ANOVA; ∗*P* < 0.05. Resting value (Rest) is following a 6 h daytime fast. Data are presented as mean ± SEM. B. Glucagon data from A. Two-way repeated measures ANOVA; ∗*P* < 0.05. n = 11 for each diet. Data are presented as mean ± SEM. C. Blood glucose following an i.p. insulin tolerance test (ITT) in mice fed a control (CTL, n = 5) or high-fat diet (HFD, n = 5). Insulin was dosed based on total body weight (0.75 U/kg). Data are presented as mean ± SEM. D. Plasma glucagon for data in *a* for 10 CTL and 11 HFD mice. Two-way repeated measures ANOVA; ∗*P* < 0.05. Data are presented as mean ± SEM. E. Blood glucose following an ITT dosed on lean mass (1.08 U/kg lean mass) in 5 CTL and 5 HFD mice. Two-way repeated measures ANOVA; ∗*P* < 0.05. Data are presented as mean ± SEM. F. Plasma glucagon for data in *c.* Two-way repeated measures ANOVA; ∗*P* < 0.05. G. Plot of blood glucose versus plasma glucagon for all *in vivo* data (including at rest, during GTT and during i.p. ITT dosed on total body weight). A single-phase decay exponential (*A exp (- a [G])*; parameters *A* and *a*; *[G]* is the plasma glucose concentration) was fit to the CTL data (R^2^ = 0.83) and HFD data (R^2^ = 0.41). The ‘half-life’ (t_1/2_) of the exponential decay is the glucose required to half glucagon secretion. For CTL, this was 1.04 (95% CI: [0.70, 1.39]) mM glucose, and for HFD, this was 2.44 [1.63, 3.71] mM glucose (N = 91 CTL mice and N = 90 HFD mice).

An increase in plasma glucose during a glucose tolerance test reduces circulating glucagon. This suppression is impaired in diabetic patients [38,39] and conditions of impaired fasting glycaemia [40,41]. HFD mice had impaired glucose tolerance and plasma glucose concentration was increased from 15 mM to 25 mM at 30 min (Figure 2A). Plasma glucagon was reduced to the same extent as in CTL mice (Figure 2B).

As glucagon is a counterregulatory hormone, we also explored whether glucagon was inappropriately secreted in HFD-fed mice during insulin-induced hypoglycaemia in response to an insulin tolerance test (ITT; Figure 2C). When insulin was dosed according to the total body weight, there was no difference in absolute glucagon (Figure 2D).

During an ITT, the majority of glucose is taken up by skeletal muscle [42]. In the ITT (Figure 2C), the insulin dose was calculated based on total body weight. However, because of the drastic difference in body composition between the diets (Table 1), this leads to an artificially high insulin dose in the HFD mice. To understand whether the higher insulin bolus in the HFD mice resulted in a greater increase in glucagon during the ITT, we also dosed insulin based on the estimated lean body mass (Figure 2E and F). Blood glucose levels were similar in the two groups 30 min after the insulin bolus, but plasma glucagon was elevated more in response to insulin in the HFD mice than in the CTL mice (Figure 2E and F), suggesting that the counterregulatory stimulation of glucagon secretion is increased in the HFD mice.

Finally, to understand how the relationship between glucose and glucagon was changed with HFD feeding, we combined all glucose and glucagon data from the ITT (dosed on total body weight) and glucose tolerance test (GTT) experiments (Figure 2G). This demonstrated that *in vivo* glucagon closely follows an exponential relationship with glucose in CTL mice (R^2^ = 0.84), with the glucose concentration required to reduce glucagon by 50% (‘half-life’) = 1.04 mM. The relationship was markedly different in the HFD-fed mice (R^2^ = 0.40), with a greater-than doubling in the glucose concentration required to suppress plasma glucagon by 50% (2.44 mM). These data suggest that glucagon is inadequately suppressed by glucose—a reported defect of TDM [8,43].

### 3.2 Intrinsic effects in islets drive the elevated plasma glucagon in the HFD-fed mice

**Figure 3:**
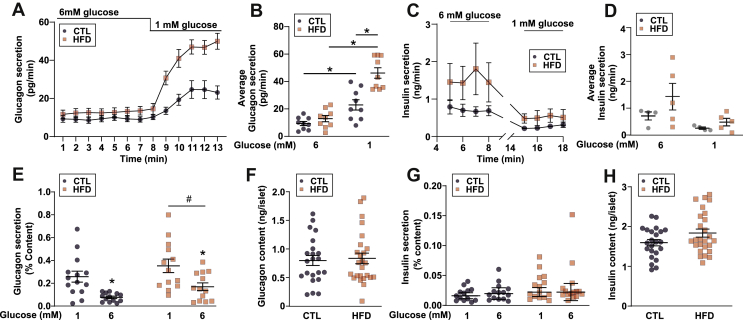
**Glucagon secretion from *ex vivo* islets or the *in situ* perfused mouse pancreas is elevated in response to HFD.** A. Glucagon measured in the perfusate of the perfused mouse pancreas from mice fed a control (CTL) or high-fat diet (HFD). n = 8–9 mice in each group. Two-way repeated measures ANOVA; ∗*P* < 0.05. Data are presented as mean ± SEM. B. Data from *a* but average steady-state values over each condition. Two-way repeated measures ANOVA; ∗*P* < 0.05. Data are presented as mean ± SEM. C. Steady-state insulin measured in the perfusate of the perfused mouse pancreas from mice fed a control (CTL) or high-fat diet (HFD). n = 4 CTL mice and n = 5 HFD mice. Two-way repeated measures ANOVA (significant source of variation: diet, *P* = 0.21; time, *P* = 0.012; interaction, *P* = 0.22). Data are presented as mean ± SEM. D. Data from c but average steady-state values over each condition. Two-way repeated measures ANOVA, *P* > 0.2. Data are presented as mean ± SEM. E. Glucagon secreted from isolated islets from CTL and HFD mice (n = 13 replicates from 6 mice). Two-way repeated measures ANOVA. Although there was no difference within a glucose concentration according to post hoc analysis, there was an overall main effect between the diets (∗*P* < 0.05). Data are presented as mean ± SEM. F. Glucagon content from isolated islets from CTL and HFD mice (n = 25 replicates from 6 mice). Unpaired *t*-test (*P* = 0.92). Data are presented as mean ± SEM. G. Same as c but insulin secretion (n = 15 replicates from 6 mice). Two-way repeated measures ANOVA; ∗*P* < 0.05. Data are presented as mean ± SEM. H. Same as *d* but insulin content (n = 24 replicates from 6 mice). Unpaired *t*-test (*P* = 0.51). Data are presented as mean ± SEM.

To determine whether the elevated plasma glucagon was due to the changes intrinsic to the islet, we measured glucagon secretion from isolated islets as well as from the *in situ* perfused mouse pancreas. In the perfused pancreas, glucagon secretion evoked by lowering plasma glucose from 6 to 1 mM was higher in the HFD mice than in the CLT mice (Figure 3A and B). The increased glucagon secretion was also observed in static incubations of isolated islets exposed to 1 and 6 mM glucose (Figure 3E and F). Finally, insulin secretion from the perfused mouse pancreas (Figure 3C and D) and isolated islets (Figure 3G and H) was (if anything) slightly (but nonsignificantly) elevated in the HFD-fed animals, making it unlikely that the hypersecretion of glucagon is due to the reduced paracrine signalling from neighbouring beta-cells.

### 3.3 Alpha-cell dysfunction is associated with altered intracellular Ca^2+^ signalling

**Figure 4:**
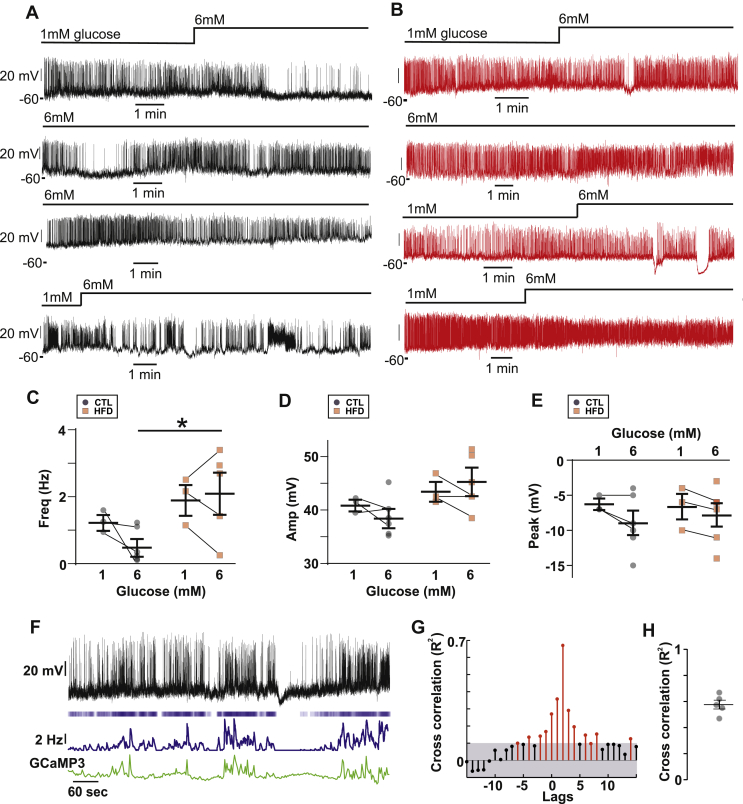
**Electrical activity in CTL and HFD alpha-cells.** A. Perforated patch-clamp recording of membrane potential (V_m_) from 4 alpha-cells from CTL mice. Note the variability in the response to high (6 mM) glucose. 6G = 6 mM glucose, and 1G = 1 mM glucose. B. Recording of V_m_ from HFD 4 alpha-cells from HFD mice. Note the variability in the response to high (6 mM) glucose. C. Firing frequency in 1 and 6 mM glucose (3–6 cells from 3 CTL mice and HFD mice). Unpaired *t*-test; ∗*P* < 0.05. Note that the lines indicate which data points are the same cell. The remaining cells were only recorded in 6 mM glucose. D. Action potential amplitude in 1 and 6 mM glucose (3–6 cells from 3 CTL and 3 HFD mice). Unpaired *t*-test, all *P* > 0.11. Note that the lines indicate which data points are the same cell. The remaining cells were only recorded in 6 mM glucose. E. Peak potential in 1 and 6 mM glucose (3–6 cells from 3 CTL and 3 HFD mice). Unpaired *t*-test, all *P* > 0.3. Note that the lines indicate which data points are the same cell. The remaining cells were only recorded in 6 mM glucose. F. Dual recording of GCaMP3 and V_m_ from an alpha-cell from a standard rodent chow-fed mouse. The upper trace is lV_m_, and below is a raster plot of action potentials with average firing frequency (calculated over a 2 s interval) and then the GCaMP3 signal from this cell. Note the correlation in average firing frequency and GCaMP3. Recording conducted in 3 mM glucose. G. Cross-correlation of GCaMP3 signal and average firing frequency. The threshold for the cross-correlation being deemed significant was R^2^ = 0.1. The lags were 1 time step (2 s). H. Maximum cross-correlation from 5 alpha-cells from 2 mice. All recordings conducted in 3 mM glucose.

**Figure 5:**
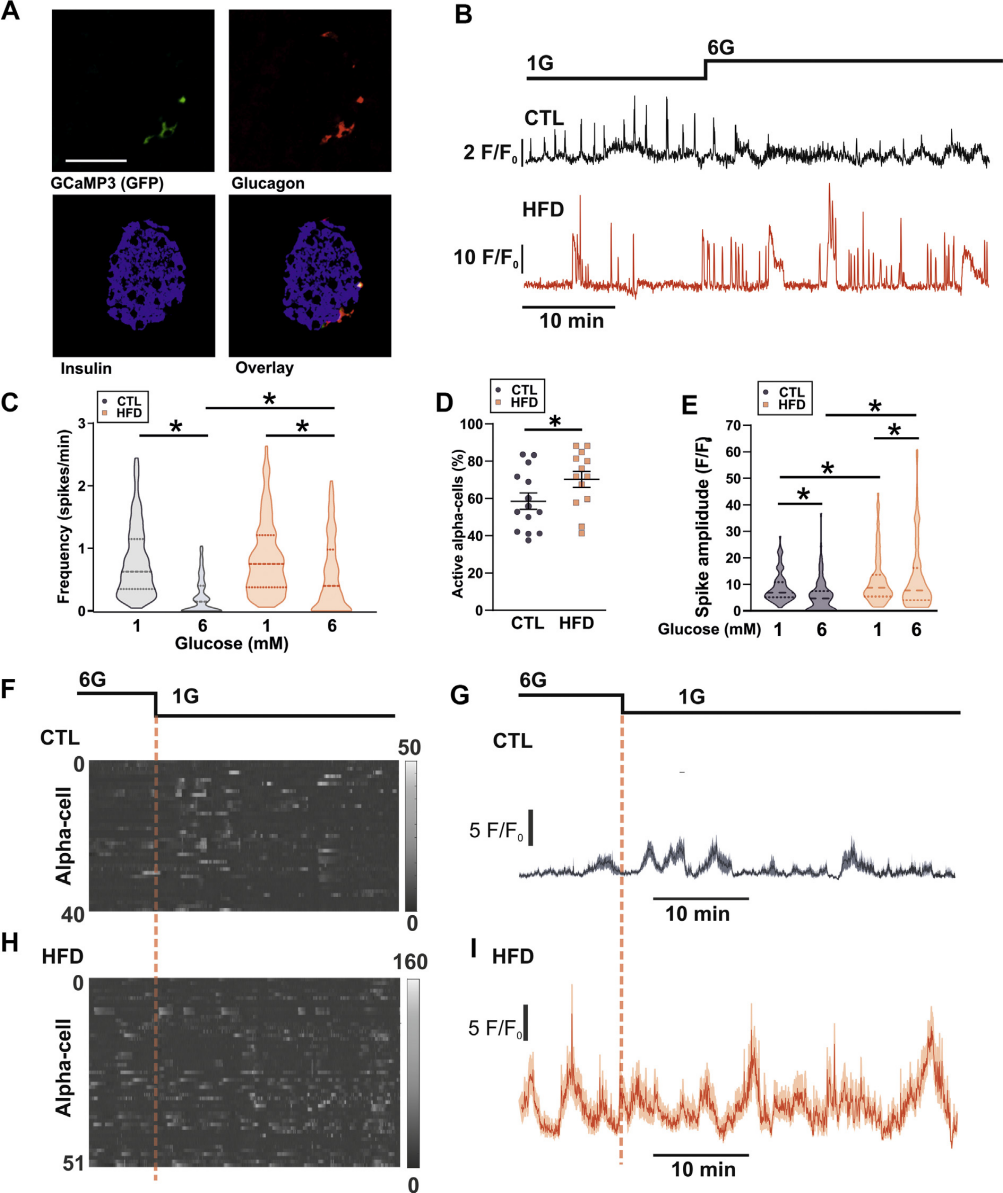
**Alpha-cells from HFD mice exhibit elevated [Ca^2+^]_i_ oscillations *ex vivo.*** A. Staining of GCaMP3, glucagon, insulin, and an overlay in islets from *Gcg*^Cre−/+^ x floxed GCaMP3 mice. Representative of 65 cells from 3 mice. The percentage of cells that were GCaMP3+ and Gcg+ was 84 ± 2%. Data are presented as mean ± SEM. B. Glucose-dependent intracellular Ca^2+^ (GCaMP3; [Ca^2+^]_i_) signals in an alpha-cell from a mouse fed a control diet (CTL) and high-fat diet (HFD). 1G = 1 mM glucose, and 6G = 6 mM glucose. C. Frequency of [Ca^2+^]_i_ oscillations in response to 1 and 6 mM glucose (n = 508 cells from 7 CTL mice, and n = 561 cells from 7 HFD mice). Data are shown as median ± quartiles. Two-way repeated measures ANOVA; ∗*P* < 0.05. Significance for HFD and CTL at 1 mM glucose is *P* = 0.092. D. Percentage of alpha-cells exhibiting [Ca^2+^]_i_ oscillations in CTL and HFD islets at 6 mM glucose. Unpaired *t*-test; ∗*P* < 0.05. Data are presented as mean ± SEM. E. [Ca^2+^]_i_ spike amplitude in response to 1 and 6 mM glucose (n = 125 cells from 4 CTL and HFD mice). Data are shown as median ± quartiles. Two-way repeated measures ANOVA; ∗*P* < 0.05. F. Raster plot of [Ca^2+^]_i_ signal from 40 alpha-cells from an islet from one CTL mouse. G. Average (±SEM) [Ca^2+^]_i_ response for all alpha-cells shown in F. H. Raster plot of [Ca^2+^]_i_ signal from 51 alpha-cells from an islet from one HFD mouse. I. Average (±SEM) [Ca^2+^]_i_ response for all alpha-cells shown in I.

Electrical activity is an important determinant of alpha-cell glucagon secretion. We first conducted patch-clamp electrophysiology of alpha-cells from CTL and HFD mice (Figure 4A and B). In the islets from both CTL and HFD mice, alpha-cells generate electrical activity at 1 mM glucose. We quantified the effects of diet on the action potential frequency (Figure 4C), amplitude (Figure 4D), and peak voltage (Figure 4E) in both 1 mM and 6 mM glucose. Of these parameters, the only statistical difference we observed was that between the action potential frequency at 6 mM glucose between the CTL and HFD mice. It is clear that the responses to glucose concentrations were variable. This same variability in the effects of glucose on the frequency and amplitude of the electrical activity in alpha-cells is evident in the literature; for example, studies report that high glucose decreases [44,45], increases [11,46], or does not change [47] action potential firing frequency in alpha-cells from wild-type mice. In our HFD alpha-cells, this may reflect the known variability in the metabolic response to high-fat feeding [48]. The perforated patch-clamp technique is challenging and has low throughput—reflected in our low sample size of cells recorded in both glucose conditions. Furthermore, it restricts the study of alpha-cells to those on the outer layer of the islet. We wanted to investigate alpha-cell function with a technique that could capture this variability with adequate statistical power. Alpha-cells exhibit oscillations in intracellular Ca^2+^ ([Ca^2+^]_i_) and changes in [Ca^2+^]_i_ drive glucagon secretion [49]. We performed parallel measurements of electrical activity and [Ca^2+^]_i_ in islets from mice expressing the genetically encoded Ca^2+^ indicator GCaMP3 under the *Gcg* promoter. We confirmed that electrical activity is correlated with [Ca^2+^]_i_ activity (Figure 4F–H). We conducted a cross-correlation of instantaneous firing frequency (calculated over a 2-second window) with the GCaMP3 signal and observed that they are highly correlated (R^2^ = 0.6, Figure 4G,H), demonstrating that [Ca^2+^]_i_ serves as a high-throughput proxy for electrical activity. We then ascertained that GCaMP3 was correctly targeted to the alpha-cells; we found that 84 ± 2% GCG^+^ cells expressed GCaMP3 (n = 3, Figure 5A). Conversely, 86 ± 7% of GCaMP3^+^ cells were GCG^+^ and only 6 ± 3% (all n = 3 mice) were INS^+^. We then fed mice expressing GCaMP3 in alpha-cells a CTL or HFD. In islets isolated from CTL mice, spontaneous [Ca^2+^]_i_ oscillations were observed at 1 mM glucose which were suppressed in frequency and amplitude when glucose was increased to 6 mM glucose (Figure 5B,C, and E). However, there was no ‘typical’ alpha-cell Ca^2+^ signature; the changes in both frequency and amplitude were extremely variable. In HFD islets, [Ca^2+^]_i_ oscillations were also observed at 1 mM glucose but these were much less affected by elevation of glucose to 6 mM (Figure 5B and C). We quantified this in a large number of CTL alpha-cells (n = 508 cells/7 mice) and HFD alpha-cells (n = 561 cells/7 mice). This analysis revealed that despite the great variability, the frequency of [Ca^2+^]_i_ oscillations was reduced by increasing glucose from 1 to 6 mM in both CTL and HFD islets (Figure 5C). However, the median frequency in 6 mM glucose was 2-fold higher in the HFD alpha-cells than in the CTL alpha-cells. Furthermore, a larger proportion of alpha-cells remained active at 6 mM glucose in islets from HFD-fed compared to the CTL-fed mice (Figure 5D). It is notable that in CTL islets ∼60% of alpha-cells remained active at 6 mM glucose (albeit at an extremely low oscillation frequency). Although glucose suppressed [Ca^2+^]_i_ oscillation amplitude in both the CTL and HFD alpha-cells, alpha-cells from the HFD-fed mice had higher spike amplitudes than those from the CTL mice at both 1 and 6 mM glucose (Figure 5E).

### 3.4 HFD islets exhibit somatostatin resistance and impaired somatostatin secretion

**Figure 6:**
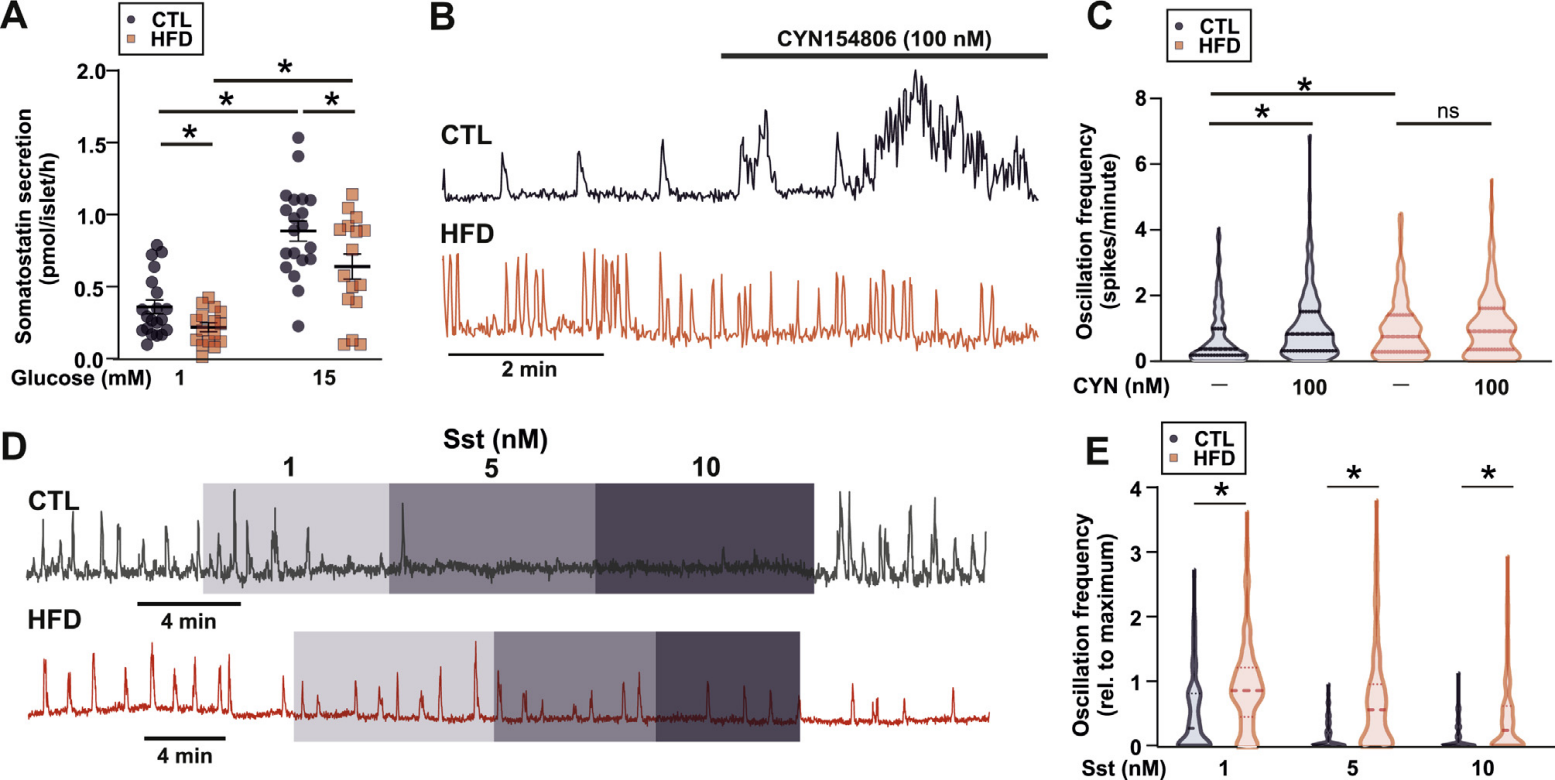
**HFD results in changes in SST secretion.** A. Somatostatin (Sst) secretion from islets isolated from control (CTL) and high-fat diet (HFD) fed mice (n = 16 replicates from 5 HFD and 5 CTL mice). *t*-test; ∗*P* < 0.05. Data are presented as mean ± SEM. B. [Ca^2+^]_i_ signal (GCaMP3) from an alpha-cell from a CTL mouse and an HFD mouse in response to the SSTR2 antagonist CYN154806 (100 nM). Recording in 6 mM glucose. C. Average alpha-cell [Ca^2+^]_i_ oscillation frequency in CTL and HFD islets in response to the SSTR2 antagonist CYN154806 (100 nM). Recording in 6 mM glucose. n = 224 cells from 5 CTL mice and n = 220 cells from 5 HFD mice. Data are shown as median ± quartiles. D. [Ca^2+^]_i_ signal (GCaMP3) from an alpha-cell from a CTL mouse and an HFD mouse in response to 1, 5, and 10 nM Sst. Recording in 1 mM glucose. E. Average alpha-cell [Ca^2+^]_i_ oscillation frequency in CTL and HFD islets in response to 1, 5, and 10 nM SST. n = 192 cells from 3 CTL mice and n = 233 cells from 5 HFD mice. Two-way RM ANOVA; ∗*P* < 0.05. Data are shown as median ± quartiles.

Alpha-cells are under strong paracrine regulation from neighbouring somatostatin-secreting delta-cells [51,52]. Long-term exposure *in vitro* of islets to the nonesterified fatty acids oleate or palmitate has been shown to reduce somatostatin (SST) secretion [25]. We, therefore, hypothesised that the increase in glucagon secretion and [Ca^2+^]_i_ oscillatory activity at 6 mM glucose may be due to lowered somatostatin (SST) secretion. Indeed, glucose-stimulated SST secretion was 30% lower in islets isolated from HFD-fed animals at both 1 mM and 15 mM glucose (Figure 6A). There was no change in the SST content between CTL (175 ± 14, 34 islets/3 mice) and HFD (193 ± 13 pg/islet, 31 islets/3 mice, *P* = 0.35) mice, nor was there a change in delta-cell number in HFD islets (Supplementary Figure 1).

To determine whether the reduced SST secretion explained the lack of inhibition of glucagon secretion by glucose, we compared [Ca^2+^]_i_ oscillatory activity in the CTL and HFD mice before and after pharmacological inhibition of somatostatin signalling in the islet. Mouse alpha-cells express primarily SST receptor 2 (SSTR2; see [7,53]). We, therefore, inhibited SST signalling using the SSTR2 inhibitor CYN154806 (CYN) and measured [Ca^2+^]_i_ oscillation frequency. At 6 mM glucose (a concentration associated with stimulation of somatostatin secretion in mouse islets; see Walker et al. [54]), the addition of CYN increased [Ca^2+^]_i_ oscillation frequency significantly in alpha-cells from the CTL mice but not in those from the HFD mice (Figure 6B and C). We also tested the capacity of exogenous SST to suppress alpha-cell [Ca^2+^]_i_ oscillation at 1 mM glucose. Whereas SST had a strong inhibitory effect in the CTL islets, the effect was much weaker in the HFD islets (Figure 6D and E). In the CTL islets, SST produced a concentration-dependent suppression of [Ca^2+^]_i_ oscillatory activity but this effect was less pronounced in the HFD islets where [Ca^2+^]_i_ oscillations persisted at the high SST concentration tested (10 nM).

**Figure 7:**
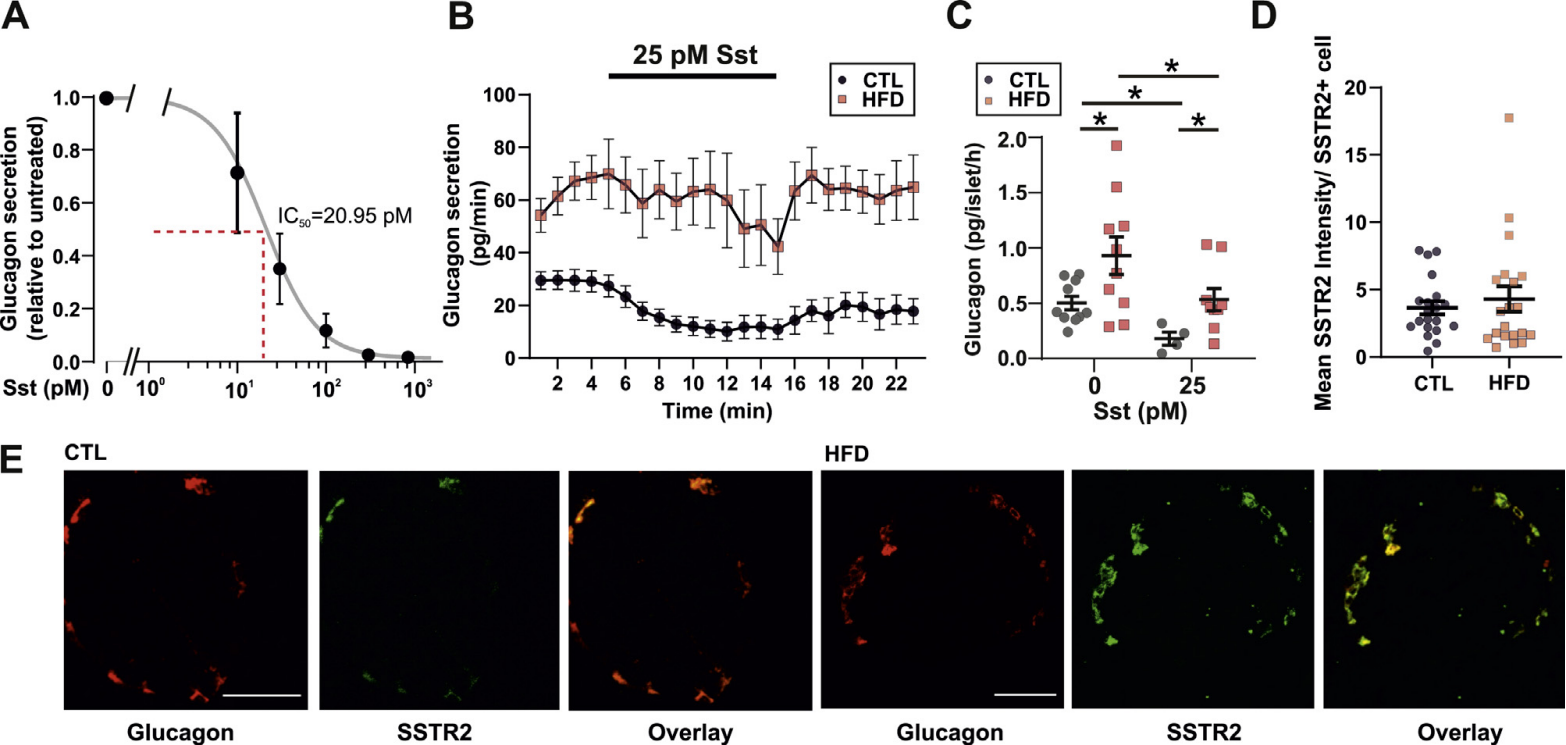
**HFD results in changes in SST resistance.** A. Dose response curve for SST on glucagon secretion measured in the perfusate from mouse pancreas in mice fed a chow diet. n = 3 mice. Data are presented as mean ± SEM. B. Glucagon measured in perfusate from CTL and HFD mice in response to 25 pM STT at 1 mM glucose. n = 9 mice in each group. Data are presented as mean ± SEM. C. Glucagon secretion from isolated islets from 3 CTL and 3 HFD mice. *t*-test, ∗*P* < 0.05. Data are presented as mean ± SEM. D. Staining of imbedded pancreata from CTL- and HFD-fed mice, for glucagon (red) and SSTR2 (green). Scale bar is 50 μm. E. Analysis of SSTR2 staining intensity from *i.* The intensity was normalised to the number of cells expressing SSTR2. n = 20 islets from 2 mice in each group. Data are presented as mean ± SEM.

Collectively, the effects of CYN154806 and exogenous somatostatin on [Ca^2+^]_i_ indicate that the alpha-cells have become resistant to SST. We further explored this possibility using the perfused mouse pancreas. We first determined the IC_50_ of SST-induced suppression of glucagon to be 21 pM in the chow-fed WT mice (Figure 7A). Accordingly, the addition of 25 pM of SST at 1 mM glucose resulted in a 60% suppression of glucagon secretion in the CTL mice (Figure 7B). In the HFD mice, glucagon secretion at 1 mM glucose was 100% higher than that in the CTL mice and the response to exogenous somatostatin was markedly curtailed with no statistically significant inhibition of glucagon secretion detected (Figure 7B). In the isolated islets, glucagon secretion in both the CTL and HFD mice was suppressed by SST (25 pM) but was higher in the HFD islets (Figure 7C).

The above findings demonstrate that, in response to HFD feeding, not only is SST secretion from delta-cells reduced but also alpha-cells become resistant to SST. We reasoned that a reduction in SSTR2 expression may underlie the observed resistance. However, SSTR2 staining in CTL and HFD pancreases revealed no differences (Figure 7D,E), in line with recent reports from obese human donors [55].

## 4 Discussion

Glucagon plays a significant role in the aetiology of diabetes [1] but little is known about how changes in alpha-cell function manifest in prediabetes. We have investigated alpha-cell function in female mice in response to prolonged ingestion of high amounts of fat (60% of total calories). We found that obese mice become hyperglucagonaemic and have impaired glucose-dependent inhibition of glucagon secretion. This echoes what is observed clinically in obese people [20,21]. While it is hard to find commonalities in the literature regarding HFD-induced hyperglucagonaemia, our findings suggest that methodological aspects such as time of sampling have a strong influence on the level of circulating glucagon measured. Thus, these findings could potentially explain why measurements of plasma glucagon in HFD-fed models differ markedly between studies [[29], [30], [31],^56^] and why it has been difficult relating them to human observations.

Glucagon secretion from alpha-cells depends on changes in [Ca^2+^]_i_. We now demonstrate that glucose regulates both the frequency and amplitude of [Ca^2+^]_i_ oscillations in alpha-cells. This is, to our knowledge, the first report distinguishing and analysing these two components of the Ca^2+^ signal and demonstrating that glucose exerts statistically significant effects on these two key parameters in the regulation of glucagon secretion that have previously escaped detection. Unlike previous studies, this study has sufficient statistical power to detect an average change despite significant cell-to-cell variability; we have analysed [Ca^2+^]_i_ in a much large number of alpha-cells (>500 for frequency and >120 for amplitude) than that analysed in earlier studies. Given that alpha-cell [Ca^2+^]_i_ and average action potential firing frequency were well correlated, the effects of HFD feeding on alpha-cell electrical activity can be extrapolated from this large [Ca^2+^]_i_ dataset. With respect to the increase in [Ca^2+^]_i_ spike frequency, one would expect that the average alpha-cell action potential firing frequency should also be increased in response to HFD feeding. Indeed, this was something we observed in our small dataset of patch-clamped cells from HFD mice. Regarding the increase in [Ca^2+^]_i_ spike amplitude in HFD-fed mice, the fact that the [Ca^2+^]_i_ signal is correlated with the average potential firing frequency suggests that alpha-cells from HFD-fed mice should exhibit more bursts of electrical activity. Given that SSTR2 activation evokes hyperpolarization of the cell membrane [57], this increase in bursting could reflect the reduction in SST secretion rather than increased SST resistance. Interestingly, not only did the activity of single alpha-cells change with HFD feeding but also the diet changed the proportion of active alpha-cells. This could account at least for some of the changes in frequency and indicates that the threshold for alpha-cell activity has been changed by HFD feeding. These findings suggest that several regulatory mechanisms underlie the changes in [Ca^2+^]_i_ and glucagon output observed with HFD feeding.

Alpha-cells are under strong paracrine regulation of SST [15,17]. A recent study suggested that delta-cell [Ca^2+^]_i_ oscillatory activity is reduced after HFD feeding [58], in keeping with the reduction of somatostatin secretion we observe. We note that the effects of HFD on somatostatin secretion—when mice remain largely normoglycaemic—are different from those observed once hyperglycaemia has developed [59]. Not only was somatostatin secretion reduced in HFD mice, but also their alpha-cells were much less sensitive to SST in the present study, with Ca^2+^ oscillation frequency persisting in HFD islet when exposed to SST. Supporting this, a recent investigation of exocytosis in >20 TDM human islet preparations of dispersed alpha-cells also observed SST resistance [60]. Our staining of Sstr2, together with other studies in human islets from obese donors [55], suggests that hyperglucagonaemia in prediabetes is not due to a reduction in SSTR2 protein. However, given the reported [60] reduction in alpha-cell SSTR2 protein expression (although a previous study [55] reported an increase in *SSTR2* mRNA in islets from T2D human donors), we suggest that the reduction in SSTR2 protein in diabetes is secondary to the as-yet-unknown primary change in SST signalling in the prediabetic state. In human alpha-cells from intact islets, a high concentration of SST (30 nM) partially (70%) inhibits glucagon exocytosis [57]. Therefore, part of the resistance to SST in the HFD alpha-cells may be due to an effect on the sensitivity of the exocytotic machinery to SST. Intestinal D-cells secrete long-form SST (SST-28), which is distinct from that which is produced and secreted by pancreatic delta-cells (SST-14; see [53,61], and [62]). As intestinal lipids stimulate GLP-1 and GIP release from the rat gut [63] and these hormones are elevated in rats fed an HFD [64], it is possible that gut SST-28 is similarly increased in response to high-fat feeding. An increase in circulating SST-28 may desensitise the alpha-cell SSTR2 receptor and/or exocytotic machinery to islet SST and result in the SST resistance we observe.

Although alpha-cells demonstrate SST resistance in response to HFD feeding and SST secretion is reduced in 1 mM glucose, this cannot fully explain the increased alpha-cell [Ca^2+^]_i_ oscillation amplitude and glucagon secretion at 1 mM glucose, where there is very little SST secretion. In rats, SST has recently been reported to inhibit glucagon secretion at 3.5 mM glucose [65]. As [Ca^2+^]_i_ amplitude was increased with HFD at 1 mM glucose—something known to be under the control of intrinsic fuel-sensing mechanisms [11,36]—we suggest that the hyperglucagonaemia present in 1 mM glucose may be driven by changes in alpha-cell metabolism. Fatty acid oxidation in alpha-cells has been shown to regulate the amplitude of [Ca^2+^]_i_ oscillations [36]. Therefore, the hyperglucagonaemia observed at 1 mM glucose in HFD alpha-cells may be due to an increase in beta-oxidation, which is an important driver of glucagon secretion [27,28].

In the current study, we did not observe SSTR2 to be located at the membrane in our mouse pancreas sections. This is in contrast to previous findings [60], which used the same antibody and demonstrated membrane localization of SSTR2 in human pancreas sections. The antibody is widely used and has been carefully validated [66]. Interestingly, SST secretion in mice is stimulated at >4 mM glucose [67]. Therefore, in living mice, where the circulating glucose concentration is 6–7.5 mM, the intraislet concentration of SST must be considerable and the ligand would be present in the preparations we have stained here. This may account for the compartmental differences in the staining of SSTR2 in alpha-cells.

The delta-cell is seemingly a key integrator and relay of circulating satiety and fuel signals to the islet alpha- and beta-cells, as it has been shown to express many GPCRs—including ghrelin receptors [68,69] and leptin receptors [70]. The NEFA-responsive G-protein-coupled receptor GPR120 is also expressed in delta-cells and its activation results in a reduction of SST secretion [71]. The elevation in circulating NEFAs in HFD-fed mice would be expected to chronically reduce the output from delta-cells, explaining the hyperglucagonaemia observed under physiological glucose concentrations. In conclusion, these findings link delta-cell dysfunction and SST resistance in alpha-cells directly to metabolic disease and demonstrated the importance of SST for the regulation of glucagon secretion in obesity and prediabetes.

## Data availability

All data are made freely available on reasonable request to the corresponding authors.

## Authors' contributions

LJBB, JGK, and JAK designed the study and experiments. LJBB and JGK wrote the initial draft of the manuscript. JAK, TGH, NJGR, SA, LJBB, and JGK conducted experiments and analysed data. All authors edited and approved the final version of the manuscript.

## Funding

This study was funded by the following: Wellcome Senior Investigator Award (095531), Wellcome Strategic Award (884655), Sir Henry Wellcome Postdoctoral Fellowship (201325/Z/16/Z), and a JRF from Trinity College. TGH is supported by a Novo Nordisk postdoctoral fellowship run in partnership with the 10.13039/501100000769University of Oxford. JAK held a D.Phil. from the OXION Programme (Wellcome). JGK held a Novo Nordisk postdoctoral fellowship run in partnership with the University of Oxford, and now receives funding from Excellence Emerging Investigator - Endocrinology and metabolism -10.13039/501100009708Novo Nordisk Fonden (0054300).

## Appendix A Supplementary data

Multimedia component 1 Cellular composition and SST content in islets from CTL and HFD mice. a. Representative staining of glucagon (red), insulin (blue), and somatostatin (green) in islets from CTL and HFD mice. b. Analysis of staining. The number of cells was counted and given as a percentage of the total number of stained cells (n = 21 (CTL) and 17 (HFD) islets from 3 mice from each diet). Data are presented as mean ± SEM. c. Islet area analysed as the stained area in each islet (n = 21 (CTL) and 17 (HFD) islets from 3 mice from each diet). Data are presented as mean ± SEM. Multimedia component 1
