# Supplementary figures and images for: Reduced somatostatin signalling leads to hypersecretion of glucagon in mice fed a high-fat diet
Source: Mol Metab. 2020 May 21;40:101021. doi: 10.1016/j.molmet.2020.101021 (PMC7322681; doi:10.1016/j.molmet.2020.101021)

# Supplementary Figure 1

a

CTL

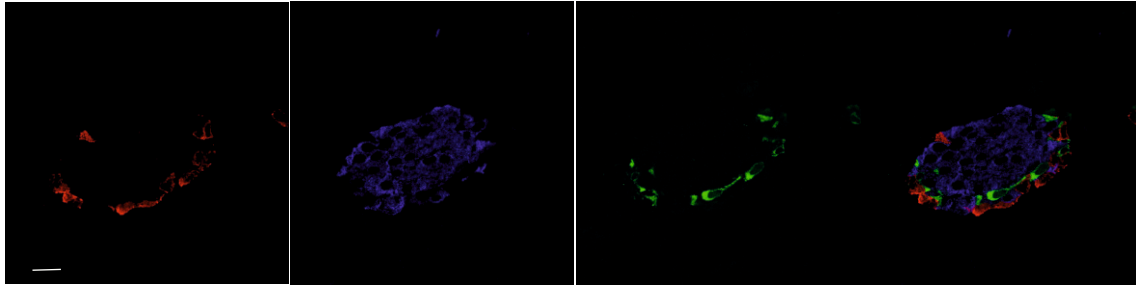

HFD

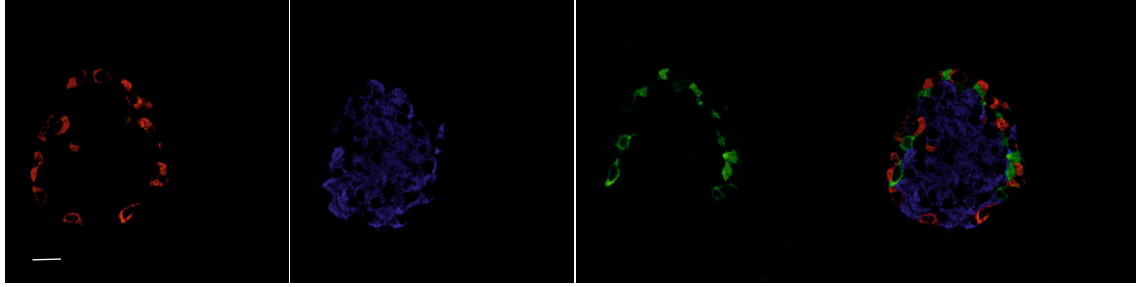

Glucagon

Insulin

Somatostatin

overlay

b

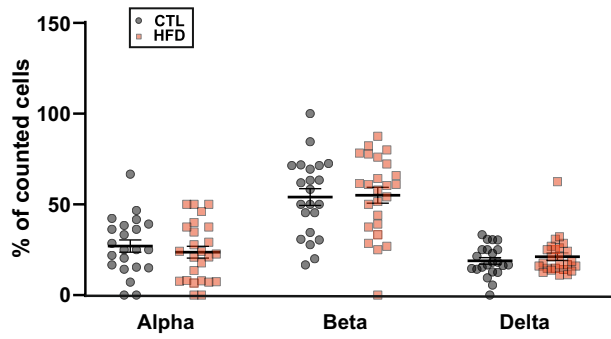

c

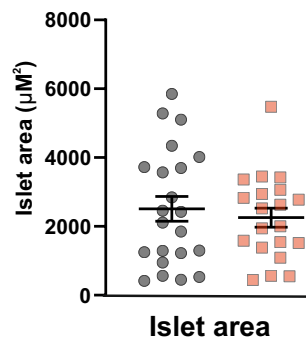

Supplement: Multimedia component 1 — Cellular composition and SST content in islets from CTL and HFD mice. a. Representative staining of glucagon (red), insulin (blue), and somatostatin (green) in islets from CTL and HFD mice. b. Analysis of staining. The number of cells was counted and given as a percentage of the total number of stained cells (n = 21 (CTL) and 17 (HFD) islets from 3 mice from each diet). Data are presented as mean ± SEM. c. Islet area analysed as the stained area in each islet (n = 21 (CTL) and 17 (HFD) islets from 3 mice from each diet). Data are presented as mean ± SEM. [file mmc1.pdf]
